# Two Cases of Tonic Pupil: Ross and Ross Syndrome Plus

**DOI:** 10.7759/cureus.22305

**Published:** 2022-02-16

**Authors:** Reyaz Ahmad, Kumar Saurabh

**Affiliations:** 1 Department of Neurology, Tata Main Hospital, Jamshedpur, IND

**Keywords:** hyperhidrosis, anhidrosis, light-near dissociation, horner’s syndrome, tonic pupil, ross syndrome

## Abstract

Ross syndrome is a rare disorder of the peripheral autonomic nervous system characterized by a triad of tonic pupils with light-near dissociation, segmental anhidrosis, and areflexia. Though having a benign course, the disease can cause significant social embarrassment. Both our cases presented with complaints of segmental facial hyperhidrosis. The first case with a one-year history had findings of segmental anhidrosis up to T4 thoracic level, left tonic pupil, and absent right ankle reflex. While the second case with a history of five years had bilateral tonic pupil, absent lower limb reflexes, anhidrosis of left face, neck, and upper trunk up to T4 level, apart from having associated Horner’s syndrome. Minor’s (starch-iodine) test and dilute pilocarpine test were helpful for diagnosis in both cases, indicating areas of anhidrosis and pupillary cholinergic denervation hypersensitivity respectively. Both cases were provided counseling and managed conservatively.

## Introduction

Ross syndrome was first described by A.T. Ross in 1958 [1]. About 60 cases have been described in literature since then [2]. It is a disorder of the peripheral nervous system characterized by a triad of the tonic pupil with light-near dissociation, areflexia, and segmental hypohidrosis. Pathogenesis hasn’t been fully elucidated but genetic predisposition with superadded environmental insults like viral infections are thought to play a role [3,4]. The course is essentially benign but features like areflexia and hypohidrosis can progress over time. Rarely apart from sudomotor and pupillary fibers other cellular subpopulations of the nervous system can also get involved resulting in features like orthostatic hypotension [5]. Though benign, the disease can cause significant social embarrassment because of segmental facial hyperhidrosis. Here we describe two cases of Ross syndrome, the second one having associated Horner’s syndrome. These cases presented at the same center within one month.

## Case presentation

Case one

A 24-year-old female presented with a complaint of increased sweating of the left side of the face and neck for one year. The patient also noted a lack of sweating on the right side of the face for three months. There was no history of urinary incontinence or urgency, postural dizziness, or postprandial abdominal fulness. The patient gave no history of cough, breathing difficulty, rashes, or joint pain. She had no history of sexually transmitted diseases (STDs). There was no significant past medical history. The patient is a non-smoker, does not use alcohol or recreational drug. A history of similar illness in the family was absent.

**Figure 1:**
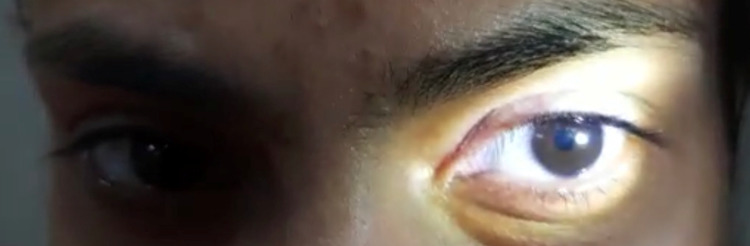
Tonic left pupil

**Figure 2:**
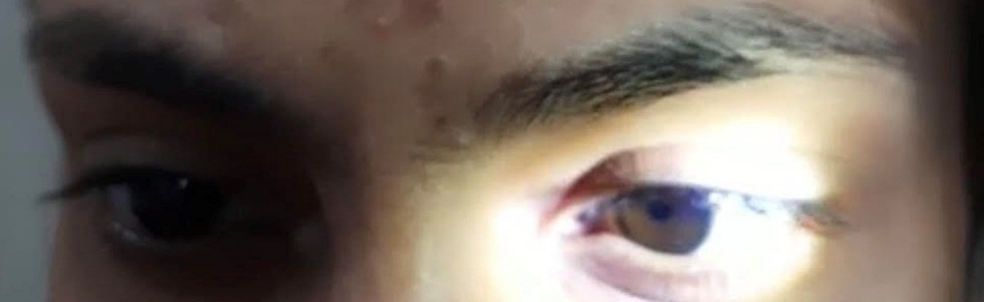
Near-reflex present

On examination, the heart rate was 78/min, supine blood pressure was 118/76 mm Hg, and standing blood pressure was 122/80 mm Hg. No abnormality was detected in the chest, cardiovascular (CVS), and abdominal examination. The central nervous system (CNS) examination revealed that higher mental functions were within the normal limit. Motor examination showed normal bulk, tone, and power in all muscle groups. Deep tendon reflexes (DTR) were absent in the right ankle and other DTR were 2+. The plantar was bilaterally flexor. Sensory examination was normal. The cerebellar examination also showed no abnormality. Cranial nerve second examinations showed normal fundus and visual fields in both eyes. Visual acuity was 6/6 meters bilaterally. The right pupil was of normal size and reactive to light. The left pupil was dilated and sluggishly reacting to light -tonic pupil (Figure 1). However, reaction to convergence (near reflex) was present indicating light-near dissociation (Figure 2). Both direct and consensual light reflexes were sluggish in the left eye (indicating third nerve involvement). All other cranial nerves examinations were normal.

Investigations

**Figure 3:**
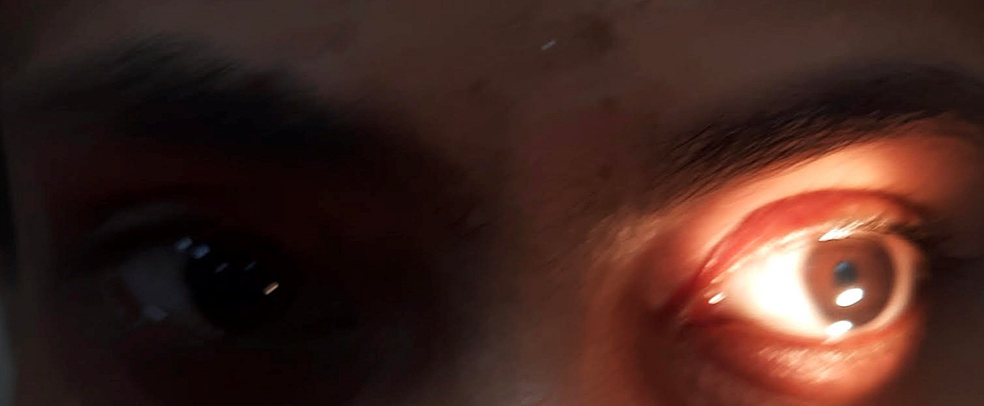
Dilute pilocarpine(0.125%) causing constriction of tonic pupil

**Figure 4:**
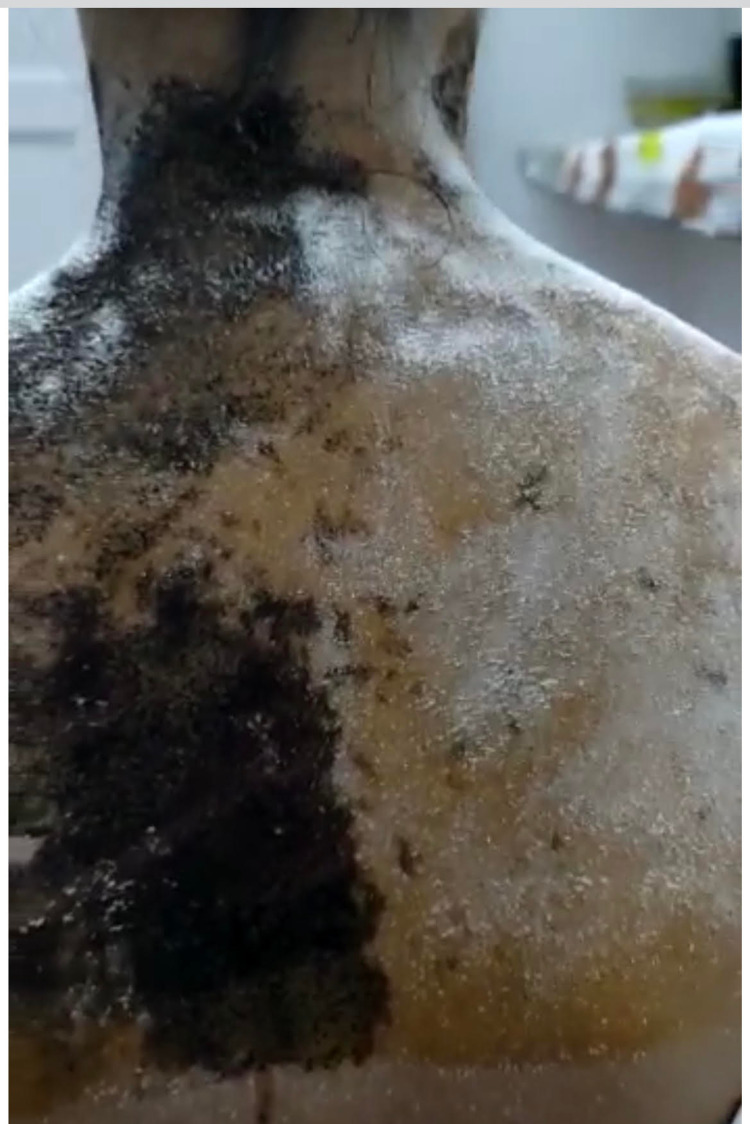
Starch-iodine test demonstrating anhidrosis - left neck and thorax

Dilute pilocarpine (0.125%) induced constriction of the tonic pupil but did not affect the normal pupil (Figure 3). Starch- iodine test showed absent sweating on the right side of the face, neck, and upper trunk up to T4 level (Figure 4). The sympathetic skin response (SSR) test was non-recordable. MRI brain and spine, chest X-ray, and ultrasound sonography (USG) abdomen were within normal limits. Refer to Table 1 for other investigations.

**Table 1:** Investigations WBC- White Blood Cells, AST- Aspartate Aminotransferase, ALT- Alanine Transaminase, RBS- Random Blood Sugar, TSH- Thyroid-Stimulating hormone, ANA- Antinuclear antibody, RPR-Rapid Plasma Reagin, HIV- Human Immunodeficiency Virus, HBsAg- Hepatitis B Surface Antigen, HCV- Hepatitis C Virus

Investigations	Patient 1	Patient 2
Hemoglobin (gm/dl)	12.2	12.2
WBC	5000	11000
Platelets	129000	255000
Serum creatinine (mg/dl)	0.77	0.8
AST/ALT (U/I)	19.3/29.4	28.3/30.5
RBS (mg/dl)	104	101
TSH (micro IU/ml)	4.71	5.53
Vitamin B12 (pg/ml)	279	1316
ANA	negative	negative
RPR	negative	negative
SS-A/SS-B	negative	negative
HIV/HBsAg/HCV	non-reactive	non-reactive

Case two

A 42-year-old female presented with complaints of increased sweating over the right side of her face for the last five years. On further questioning, she mentioned absent sweating over the left side of the face. There was no history of urinary incontinence or urgency, postural dizziness, or postprandial abdominal fulness. Also, no history of cough, breathing difficulty, rashes, joint pain, or recurrent fetal abortion was found. The patient gave no history of STD. She had a history of hypothyroidism for the last six years. There is no history of similar illnesses in family members.

**Figure 5:**
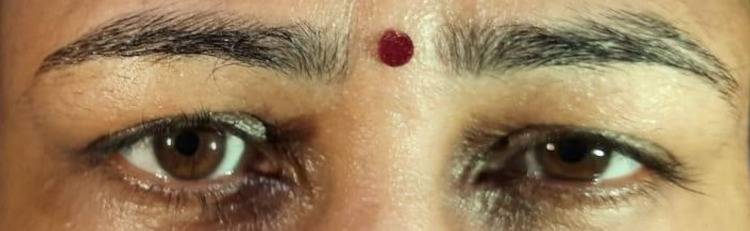
Bilateral tonic pupil with smaller left palpebral fissure and inverse ptosis (Horner’s syndrome) in case 2

On examination, heart rate was 74/min. Supine and standing BP was 138/88 and 116/76 mm Hg respectively. No abnormality was detected in the chest, CVS, and abdominal examinations. CNS examination showed higher mental functions were within normal limits. Motor examination showed normal bulk, tone, and power in all muscle groups. Deep tendon reflexes were normal in upper limbs, however bilateral knee and ankle reflexes were absent. Plantar reflex was bilaterally flexor. Sensory examination showed no abnormality. The cerebellar examination was also within the normal limit. Cranial nerve second examination revealed fundus, visual fields, and acuity (bilateral 6/6 meters) were within normal limits. The right and left pupils were 5 mm and 6 mm dilated respectively. Both pupils were sluggishly reacting to light. However, pupillary constriction was present with a near reflex (light-near dissociation). Direct and consensual light reflexes were sluggish in both eyes. Third, 4th, and 6th cranial nerve- extraocular movements were normal. The left palpebral fissure was smaller than the right one, with evidence of inverse ptosis indicating Horner’s syndrome. All other cranial nerves examinations were normal (Figure 5).

Investigations

**Figure 6:**
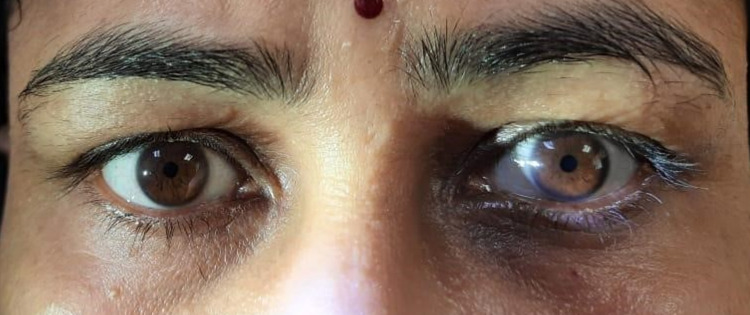
Dilute pilocarpine (0.125%) causing constriction of both tonic pupil

**Figure 7:**
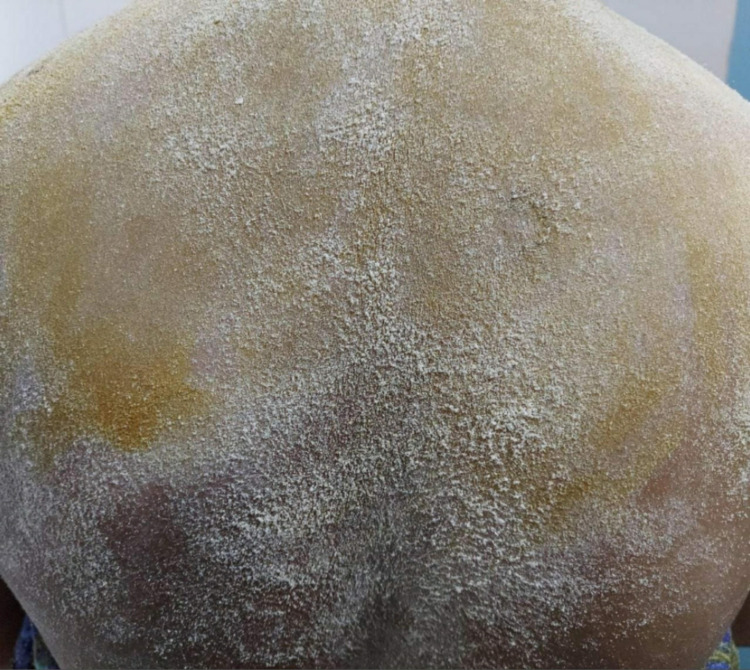
Starch- iodine test in case 2

Dilute pilocarpine (0.125%) induced constriction of the tonic pupil in both eyes (Figure 6). Starch-iodine test showed absent sweating on the left side of the face, neck, and whole upper trunk up to T4 level, with some areas of retained sweating in the anterior and lateral left shoulder (Figure 7). SSR test was non-recordable. MRI brain and spine, chest x-ray, and USG abdomen were within normal limits. Refer to Table 1 for other investigations. 

## Discussion

Both the patients presented with a triad of the tonic pupil with light-near dissociation, segmental anhidrosis with compensatory hyperhidrosis, and areflexia. This combination of signs and symptoms point to the involvement of the autonomic nervous system with differentials of Ross syndrome, Holmes-Adie syndrome, and Harlequin syndrome. In Holmes-Adie syndrome there is no involvement of the sudomotor system and in Harlequin syndrome pupils are spared. Hence a diagnosis of Ross syndrome was kept in both of our cases. However, few studies have shown subclinical sudomotor and pupillary involvement in Holmes-Adie and Harlequin syndrome respectively. There are suggestions that Ross, Holmes-Adie, and Harlequin syndromes are different manifestations of the same disease process [5-8].

Ross syndrome is a rare peripheral autonomic system disorder with the predominant involvement of postganglionic sudomotor and pupillary fibers. Theories regarding its pathogenesis include genetic susceptibility with superadded environmental insult. One case series reported Ross syndrome cases in identical twins [3]. Others have reported disease development after infections like Cytomegalovirus (CMV) [4]. A recent case series proposed that Ross syndrome can be a synucleinopathy like Parkinson’s disease, Dementia Lewy body, Multiple System Atrophy, and Pure Autonomic Failure [9]. Some case series and reports have found an occasional association between autoimmune diseases like Sjogren syndrome and Ross syndrome [10]. Few others reported associated antinuclear antibody (ANA) positivity [11]. In both of our cases, there were no clinical features of antibody profiles suggestive of this association.

The tonic pupil is due to damage to ganglionic and post-ganglionic cholinergic fibers of ciliary ganglion. Other causes of tonic pupil like Holmes- Adie syndrome (no sudomotor involvement), second nerve [associated with relative afferent pupillary defect (RAPD)], and third nerve (associated with ophthalmoplegia and ptosis) palsy also needs to be excluded [12]. In the late stages, the tonic pupil becomes miotic when it needs to be distinguished from Horner’s syndrome. In our first case, a tonic pupil was present in the left eye, while the right eye pupil was normally reactive to light. Though Ross syndrome commonly presents with bilateral involvement of pupils as against predominant unilateral involvement seen in Holmes-Adie syndrome, early cases can present with unilateral pupillary involvement [2]. In our second case, pupillary involvement was bilateral.

Light-near dissociation in Ross syndrome is caused by aberrant regeneration of fibers to pupils which were originally destined for ciliary muscles, as parasympathetic fibers for ciliary muscles greatly outnumber those going to pupillary muscles. Other causes of light-near dissociation include Argyll- Robertson pupil and Parinaud syndrome (dorsal midbrain syndrome). In these cases, light-near dissociation is due to selective involvement of dorsally located light reflex pathway in the midbrain with sparing of the ventral near-reflex pathway [12].

Anhidrosis in Ross syndrome is generally segmental to start with, which can later become generalized. Hyperhidrosis is mainly compensatory in Ross syndrome. However other theories suggest that involvement of preganglionic muscarinic M2 receptors leading to increased acetylcholine release in the early stages of the disease can also be a possibility [13]. In our first case, anhidrosis was present in the left side of the face, neck, and thoracic area up to T4 level. In our second case sweating was absent over the left side of the face, neck, and upper trunk up to the level of T4 with few islands of retained sweating in the left shoulder area indicating advanced disease with widespread sudomotor disturbance.

Hyperhidrosis was the presenting complaint and main point of concern for both the patients. Many studies also point towards the fact that hyperhidrosis is the predominant distressing complaint in patients of Ross syndrome as it is more likely to cause social embarrassment. However, anhidrosis if widespread can lead to serious complications like heat-stroke apart from the usual complaint of heat intolerance. According to one study hyperhidrosis was the presenting complaint in the majority of patients followed by anhidrosis and complaints related to tonic pupil [2]. Cases presenting with segmental hyperhidrosis or anhidrosis should be screened for pupillary and muscle stretch reflex abnormalities to look for the typical triad of Ross syndrome.

Hyporeflexia is due to the involvement of neurons in dorsal root ganglia (DRG) and spinal interneurons. One hypothesis is that DRG neurons and parasympathetic nervous systems have similar origins from neural crest cells, explaining DRG neurons involvement in Ross syndrome patients [14]. Our first case had absent right ankle reflex, while the second case had bilaterally absent knee and ankle reflex.

Various studies have demonstrated benign course of Ross syndrome patients. However, areas of hypohidrosis can progress to anhidrosis and the initial segmental pattern of hypohidrosis can enlarge to become generalized. Hyporeflexia or areflexia can also progress with time [5]. Our first case has a one-year history of segmental anhidrosis, whereas the second case with a history of five years had bilateral anhidrosis with patches of intact sweating in anhidrotic areas. In our first case, only the right ankle reflex was absent, but the second case had areflexia at both the knees and ankles. Also, the first case had unilateral pupillary involvement, but in the second case, pupillary involvement was bilateral.

Apart from pupillary and sudomotor involvement, other nervous system areas can also become involved with time. Development of orthostatic hypotension and subclinical sensory system involvement has been demonstrated in some case series [5]. Our second case though not having complaints of postural dizziness showed significant postural variation in blood pressure. 

Few cases of Ross syndrome associated with Horner’s syndrome have been described in the literature [7]. Our second Ross syndrome case was associated with Horner’s syndrome. Both syndromes present in the same patient indicate a wider involvement of the autonomic nervous system.

Treatment is mainly symptomatic. For heat intolerance, the patient is advised to avoid heavy activities in a warm climate and wear wet clothes. Hyperhidrosis can be treated by local application of glycopyrrolate and aluminum chloride cream [15]. Botulinum injection can also be used but can cause muscular weakness [16]. Thoracic sympathectomy can be an option for severely symptomatic patients who are willing to undergo an invasive procedure [17]. Apart from this adequate counseling about the benign nature of the disease should also be done for this socially embarrassing disease. Patients should be followed for progression and involvement of other nervous system areas. Both of our cases were counseled about activity modification and managed conservatively.

## Conclusions

Ross syndrome is a rare disorder of the peripheral autonomic nervous system that presents with a triad of the tonic pupil, anhidrosis, and areflexia. Pilocarpine test, starch-iodine test, and SSR are helpful in diagnosis. Our second case was associated with Horner’s syndrome indicating widespread autonomic nervous system involvement. Though benign, the disease can be socially crippling. So, patients need to be properly counseled. Finding two cases from a single center within a month is an indication that Ross syndrome might not be as rare as reported in the literature.
